# How and when seeking feedback from coworkers pays off? The mixed role of coworker relationship

**DOI:** 10.3389/fpsyg.2022.938699

**Published:** 2022-08-16

**Authors:** Wei Zhang, Jing Qian, Haibo Yu

**Affiliations:** ^1^School of Government, Beijing Normal University, Beijing, China; ^2^Business School, Beijing Normal University, Beijing, China

**Keywords:** coworker feedback-seeking behavior, coworker relationship, task performance, workplace well-being, social exchange theory

## Abstract

Although research in the feedback-seeking behavior literature has primarily focused on feedback-seeking from supervisors, some emerging works have begun to explore the benefits of coworker feedback-seeking behavior. Based on the social exchange theory, we investigated how and when seeking feedback from coworkers will benefit the seekers. Using a sample of 327 teachers from China, we find that seeking feedback from coworkers is positively associated with task performance and workplace well-being. Seeking feedback from coworkers is also positively associated with coworker relationship. Moreover, the coworker relationship mediates the effects of seeking feedback from coworkers and task performance and workplace well-being, and moderates the strength of the relationship between seeking feedback from coworkers on task performance and workplace well-being. Theoretical and practical implications of our findings are discussed.

## Introduction

Feedback is a valuable resource for employees to modify their behaviors and improve their performance (Ashford, 1986). To obtain this resource, individuals not only passively wait for formal performance appraisal but also actively seek feedback from various targets (e.g., their leaders, colleagues, and environment) (Ashford et al., 2003). Most previous studies have explored feedback-seeking behavior in the supervisor–subordinate dyads, where the subordinates act as the seeker and supervisors act as the target of feedback (Ashford et al., 2016). This is because leaders always have multiple resources and are empowered to assign rewards and punishments (Ashford, 1993). However, seeking feedback from coworkers received little research attention (Ashford et al., 2016). In fact, coworkers can be important information sources and referents (Takeuchi et al., 2011), and their feedback has special advantages and sometimes can complement the feedback from leaders. For example, coworkers are more familiar with the specific knowledge of one's work than leaders, as they are doing similar tasks (De Stobbeleir et al., 2020). Despite the efforts on establishing the direct positive link between seeking feedback from coworkers and individual- and team-level performance (Wu et al., 2014; De Stobbeleir et al., 2020), scholars have largely overlooked the underlying mechanism of this behavior (for an exception: Whitaker et al., 2007). While Whitaker et al.'s (2007) only focused on the cognitive mechanism in this relationship, other potential mechanisms are still void. Exploring how and when coworker feedback-seeking behavior plays a role is important because doing so helps understand how peer feedback-seeking could benefit most.

To solve this problem, we draw upon social exchange theory (Blau, 1964) and suggest that coworker relationship can help us understand how and when seeking feedback from coworkers pays off. Notably, feedback-seeking behavior involved social interaction (i.e., seeking and giving feedback) and was constrained by social context (Whitaker and Levy, 2012; Xing et al., 2021). While social exchange theory proposed that high-quality relationship was derived from some positive perceptions, such as obligation, trust, and gratitude, as well as respect, contributions, and liking (Blau, 1964; Liden and Maslyn, 1998; Gables et al., 2014), we propose a positive relationship between coworker feedback-seeking behavior and coworker relationship. This is because seeking feedback from coworkers not only helps individuals gain information and advice about how to perform their work (i.e., the instrumental value) (Lam et al., 2017) but also elicits a sense of mutual trust and support through interpersonal communication (i.e., the affective value) (Methot et al., 2021).

Moreover, we argue that high-quality coworker relationship will in turn enhance individuals' task performance (i.e., the proficiency with which employees carry out the core requirements on the job, Lee et al., 2021, P. 81) and workplace well-being (i.e., job satisfaction and positive emotions toward one's work, Zheng et al., 2015). We especially focused on these two outcomes because coworker relationships have mixed instrumental and affective effects (Chen and Peng, 2008). In detail, the instrumental effect is more work-related, manifested as mutual trust and mutual assistance at work (Chen and Peng, 2008), thereby affecting individuals' task performance; moreover, the affective effect is more nonwork-related (Chen and Peng, 2008), and has the potentials to generate positive feelings, we thus explore its impact on well-being.

Meanwhile, because coworker feedback-seeking behavior and coworker relationship have similar functions (i.e., the instrumental value and the affective value) (Chen and Peng, 2008; Whitaker and Levy, 2012), we argue that these two factors compensate and compete with each other when they play roles at the same time. Because feedback-seeking behavior is an important way to gain resources (Ashford, 1986) and the resource gains are more evident in the context of resource loss or a lack of resources (Halbesleben et al., 2014; Hobfoll et al., 2018), coworker relationship and coworker feedback-seeking behavior will compensate less and compete more in a state of abundance of resources and will compensate more and compete less in a state of scare resources. We, therefore, expect that coworker feedback-seeking behavior will be more positively correlated with task performance and workplace well-being in high-quality coworker relationships. That is, employees with high-quality coworker relationships may experience little enhancement from coworkers' feedback-seeking behavior, as they have already gained abundant feedback and affective support from coworkers (Sherony and Green, 2002; Anand et al., 2010).

**Figure 1:**
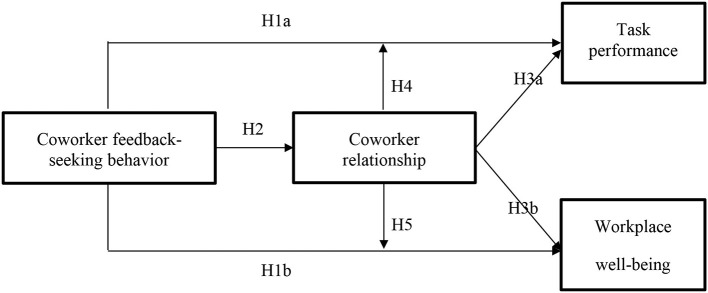
The theoretical model.

This study has several contributions. First, we contribute to coworker feedback-seeking behavior literature by providing one possible mechanism. While extant research has established the direct effect between peer feedback-seeking behavior and performance (Wu et al., 2014; De Stobbeleir et al., 2020), as well as explored the underline mechanism from a cognitive perspective, we know little about alternative mechanisms of this behavior. Moving on this, we investigated how coworker relationship transformed the function of peer feedback-seeking behavior. Second, we captured the social exchange nature of feedback-seeking process and explored the dual roles of the coworker relationship in feedback-seeking behavior. Prior research has concluded the bright and dark side of relationship quality; thus, we go further and prove the compensatory and competing effect of coworker relationship with coworker feedback-seeking behavior in one theoretical model. Finally, we contribute to the literature on the consequences of feedback-seeking behavior. While prior research proposed that social interactions are indicators of individuals' well-being, fewer studies have explored the impact of feedback-seeking behavior, which contains social factors, on individuals' well-being (Sonnentag, 2015; Ashford et al., 2016). We extend this research line by establishing and examining the coworker feedback-seeking behavior and well-being relationship. Figure 1 depicts our theoretical model.

## Theory and hypotheses

### The mediating role of coworker relationship

Feedback-seeking behavior is defined as “the conscious devotion of effort toward determining the correctness and adequacy of behaviors for attaining valued end states (Ashford, 1986, P. 466).” It has instrumental values for individuals to meet their goals and regulate their behaviors (Ashford et al., 2003). As individuals spend most of their time with coworkers and most tasks require the cooperation of team members, individuals can thus benefit a lot from seeking feedback from coworkers (De Stobbeleir et al., 2020). In line with this, we propose a positive relationship between seeking feedback from coworkers and task performance.

On the one hand, individuals can obtain more specific feedback information from coworkers because they are on the same level and doing similar work tasks, which allows coworkers to better understand the details of their work (Kim and Yun, 2015). For example, when a student is trying to determine if there is a problem with the way he/she used to download a research paper, asking his/her peers rather than the supervisor can get more detailed information. On the other hand, as tasks become more interdependent, seeking feedback from coworkers helps individuals understand the progress and direction of their tasks, which provides cues for regulating their behaviors and in turn improves job performance (De Stobbeleir et al., 2020). To support these views, research has indicated that seeking positive feedback about peers' performance was positively related to individuals' job performance (Gong et al., 2017). Accordingly, we hypothesize the following:

***Hypothesis 1a:*** *Feedback-seeking from coworkers is positively related to task performance*.

Although feedback-seeking behavior has instrumental value for job performance, it is interactive by nature (Williams et al., 1999). Research has indicated that positive social interactions such as support from coworkers are predictors of well-being (Sonnentag, 2015). As feedback-seeking behavior is “in essence socially constructed” (Ashford et al., 2016, P. 226), and involves effective interactions between feedback seekers and feedback sources, we argue that seeking feedback from coworkers is positively related to workplace well-being.

Indeed, the interactive dialogues embedded in feedback-seeking behavior can help establish connections and mutual trust between the feedback seeker and the feedback source (i.e., coworkers) (Methot et al., 2021). In support of this view, research has indicated that daily small talk with coworkers generated positive social emotions, including friendly and close feelings (Methot et al., 2021). In addition, research has indicated that individuals could reduce uncertainty by seeking feedback from others (Ashford and Cummings, 1983). Taken together, the increased connection and trust, as well as the decreased perceptions of uncertainty, will contribute to an individual's workplace well-being (Sonnentag, 2015). Accordingly, we hypothesize the following:

***Hypothesis 1b**: Feedback-seeking from coworkers is positively related to workplace well-being*.

As peer feedback-seeking behavior is labeled as “relational proactivity,” which aims to create, maintain, and improve relationships (De Stobbeleir et al., 2020), we expect that seeking feedback from peers will be positively related to coworker relationship. In detail, a high-quality coworker relationship is characterized by obligation, trust, and respect and it can be developed through effective social exchange (Sherony and Green, 2002). As seeking feedback from coworkers can provide individuals with valuable information and psychological benefits (e.g., a sense of connection), it will help improve the relationship among colleagues (Lam et al., 2017; Methot et al., 2021). To support this view, results from a meta-analysis have indicated a positive relationship between feedback-seeking behavior and relationship-building (*r* = *0.27*) (Anseel et al., 2015). Accordingly, we hypothesize the following:

***Hypothesis 2*** *: Feedback-seeking from coworkers is positively related to coworker relationship*

A close coworker relationship was indicated by “higher levels of trust and positive affect” (Chen and Peng, 2008, P. 64). In this vein, the coworker relationship has both instrumental and affective functions (Chen and Peng, 2008). On the one hand, with the increasing reliance among colleagues, higher levels of relationship help them cooperate well with each other and get the work done (i.e., the instrumental function) (Chen and Peng, 2008). On the other hand, the close relationship among coworkers can arouse positive feelings toward them (i.e., the affective function) (Chen and Peng, 2008). Therefore, the instrumental function of the coworker relationship will be helpful in performance improvement, while the affective function will increase their workplace well-being. Consistent with this argument, recent research has explored the important role of coworkers in individuals' job performance and found that coworker support and coworker exchange were positively related to psychological flourishing and employee performance (Singh et al., 2019).

Combined with the above hypotheses (i.e., Hypothesis 1a–Hypothesis 2), we further propose the mediating role of the coworker relationship in the relation between seeking feedback from coworkers and task performance (i.e., Hypothesis 1a) and workplace well-being (i.e., Hypothesis 1b). Accordingly, we hypothesize the following:

***Hypothesis 3a:*** *Feedback-seeking from coworkers is positively related to task performance through increased coworker relationship*.
***Hypothesis 3b:*** *Feedback-seeking from coworkers is positively related to workplace well-being through increased coworker relationship*.

### The moderating role of coworker relationship

Social exchange theory proposes that the levels of relationship quality determined the exchange patterns among individuals (Blau, 1964). Similar to leader–member exchange (i.e., LMX), which indicated that leaders developed different relationships with their subordinates (Martin et al., 2016), coworker relationship signified that employees exchanged differently with their coworkers on a lateral level (Sherony and Green, 2002). A high-quality coworker relationship is manifested as extended social exchange, including mutual trust and positive affection (Chen and Peng, 2008), whereas a low-quality coworker relationship is characterized by limited social exchange (Sherony and Green, 2002).

In the context of high-quality coworker relationships, employees tend to communicate frequently and exchange valuable resources (e.g., tacit knowledge, and emotional support) with coworkers (Zhang et al., 2021). The resources (i.e., instrumental and affective values) obtained from coworkers' social exchange will induce increased task performance and workplace well-being. In support of these views, research has indicated that diagnostic feedback is positively related to job performance (Ashford et al., 2016) and interpersonal interactions are important predictors of workplace well-being (Sonnentag, 2015). In this regard, employees in high-quality coworker relationships are in a more resourceful state (i.e., both including instrumental and affective resources) than those in low-quality coworker relationships.

We contend that the quality of coworker relationship may discourage employees from experiencing the benefits of coworkers' feedback-seeking. Employees with high-quality coworker relationships have already possessed abundant instrumental and affective resources to improve their performance and well-being. Thus, coworker feedback-seeking behavior may have less impact on task performance and workplace well-being in this context (i.e., high-quality coworker relationships). By contrast, colleagues in low-quality relationships communicate less frequently and are less likely to spontaneously provide support or task knowledge (Sherony and Green, 2002); employees in this context tend to possess limited instrumental and affective resources that can be used to promote their performance and well-being. In this vein, the relationship between coworker feedback-seeking behavior and task performance and workplace well-being will turn stronger when employees with low-quality coworker relationships gather resources from seeking feedback. Taken together, while feedback-seeking behavior and coworker relationship quality are widely assumed as positive impactors, we argue that they compensate and compete with each other when they play roles simultaneously. Specifically, in high-quality coworker relationships, these two effects compensate less and compete more with each other, and in low-quality coworker relationships, these two effects compensate more and compete less with each other. This is because “resource gains will take on greater meaning in the context of resource loss (or lack of resources) (Halbesleben et al., 2014, P. 1335),” and the high-quality coworker relationships represent abundant resources whereas the low-quality relationships represent a lack of resources.

To support our arguments, research has demonstrated the detrimental role of LMX in feedback-seeking behavior from supervisors, and suggested that individuals gained less (i.e., low-level performance) from this behavior in a high-LMX context (Lam et al., 2017). Likewise, relationship qualities with leaders and teammates have been found to minimize the potential benefit of i-ideals (i.e., one form of proactive behavior). That is, employees who have a high-quality relationship with their leaders and teammates already feels appreciated in the workplace, which will experience less enhancement through i-ideal (Anand et al., 2010). Accordingly, we hypothesize the following:

***Hypothesis 4:*** *Coworker relationship moderates the positive relationship between feedback-seeking from coworkers and task performance such that the relationship is weaker when coworker relationship is high (vs. low)*.
***Hypothesis 5:*** *Coworker relationship moderates the positive relationship between feedback-seeking from coworkers and workplace well-being such that the relationship is weaker when coworker relationship is high (vs. low)*.

## Materials and methods

### Participants and procedure

Data were collected from 22 kindergartens located in northern China. We collected our data based on a Talent Assessment Project, which aimed to build a competence model for the teachers in this region. With the help of the directors, we established 22 WeChat (i.e., a popular instant messaging app in China) groups and distributed online questionnaires to the kindergarten teachers. Participants were asked to report their demographic information, coworker feedback-seeking behavior, coworker relationship, task performance, and workplace well-being. The questionnaires were distributed by an online link, and research assistants reminded those who forgot to fill out the questionnaire within one week. Participants completed the questionnaire voluntarily, and their responses were assumed to be confidential.

The number of teachers participating in the Talent Assessment Project was 600. We initially distributed our questionnaires to all of the teachers, of which 546 responded (response rate: 91%). After excluding invalid questionnaires (the answering time was too short, or the same answer was chosen for all measurement items), a total of 327 valid questionnaires were obtained.

Of the final 327^1^ teachers, 96% were women (SD = 0.20), the average age was 34.32 (SD = 9.80, ranging from 21 to 56). About 83.5% of them hold a bachelor's degree, 15.6% hold a junior college degree, and 0.3% and 0.6% of them hold a master's and high school degree, respectively. Most of them (96%) came from public-established kindergartens and the others (4%) were from private-established kindergartens.

### Measures

All measurement items were originally developed in English and then translated into Chinese by the back-translation procedure (Brislin, 1980). Unless especially mentioned, we use a five-point Likert scale with 1 = “strongly disagree” to 5 = “strongly agree” to measure all the variables.

#### Coworker feedback-seeking behavior

We used a seven-item scale developed by Callister et al. (1999) to measure coworker feedback-seeking behavior. Participants were asked to rate how often they engaged in corresponding behaviors (1 = “none” to 5 = “frequently”). The sample items were “I ask my coworkers if I am doing a good job” and “From their (my coworkers') reactions, I can tell how well I am getting along with members of my work group.” The Cronbach's α for the scale was 0.90.

#### Coworker relationship

We measured coworker relationship using a nine-item scale developed by Chen and Peng (2008). The sample items were “We support each other at work” and “We trust each other.” The Cronbach's α for the scale was 0.93.

#### Task performance

We employed a five-item scale developed by Bachrach et al. (2007) to assess task performance. A sample item was “Adequately complete assigned duties.” The Cronbach's α for the scale was 0.96.

#### Workplace well-being

We used a six-item scale developed by Zheng et al. (2015) to measure workplace well-being. A sample item was “I am satisfied with my work responsibilities.” The Cronbach's α for the scale was 0.94.

### Control variables

Following previous research, we controlled for demographic variables (i.e., gender, age, education) as they have potential effects on individual task performance and workplace well-being (Bachrach et al., 2007; Zheng et al., 2015). As for the demographic variables, gender was coded 1 as “male,” and 2 as “female”; age was measured by the number of years; and education level was coded 1 as “high school,” 2 as “junior college,” 3 as “undergraduate,” 4 as “master,” and 5 as “doctor.”

### Analytic strategy

We first conducted Harman's single-factor test to examine whether the substantial variance of our data would be accounted for by one single factor, as our data were rated by the same source. Second, we conducted a confirmatory factor analysis (CFA) to examine the fit of our four-factor model (i.e., coworker feedback-seeking behavior, coworker relationship, task performance, and workplace well-being). Finally, we tested our hypotheses in Mplus 8.3 (Muthén and Muthén, 2017) by running three models. Specifically, the first model (M1) was used to test the direct effect of coworker feedback-seeking behavior on task performance and workplace well-being. The second model (M2) was conducted to test the mediating effects of coworker relationship, using the MODEL CONSTRAINT command in Mplus. Finally, the third model (M3) estimated all paths simultaneously to test the moderating effects of coworker relationship.

## Results

### Accessing common method bias

Harman's single-factor test was conducted to evaluate whether our hypothesized relationships might be disturbed by common method bias. We applied a principal component factor analysis to all the items of our studied variables, extracting six factors, and the first factor accounted for 36.27% of variance (falling below the recommended criteria of 40%). These results suggested that the common method bias of our study was not a serious problem.

### Discriminant validity and descriptive statistics

We conducted a confirmatory factor analysis to examine the distinctiveness of the studied variables. Following Landis et al. (2000), we created three-item parcels per construct to reduce the sample size-to-parameter ratio. Specifically, as all the studied variables were not unidimensional, we applied the domain-representative approach (i.e., combining items across facets into a parcel) to form parcels for the latent variable which was multidimensional (Williams et al., 2009). As shown in Table 1, the four-factor model (χ^2^ = *74.53, df* = *48, CFI* = *0.99, TLI* = *0.99, RMSEA* = *0.041*) has a better fit than the other four alternative models, indicating the good distinctiveness of our measurement.

**Table 1:** Results of confirmatory factor analysis.

**Model**	**Factors**	**χ^2^**	**df**	**CFI**	**TLI**	**RMSEA**
Null model		2,963.71	54	0.33	0.18	0.406
Baseline model	Four factors	74.53	48	0.99	0.99	0.041
Model 1	Three factors: combine CFSB and coworker relationship	1,078.15	51	0.76	0.69	0.248
Model 2	Three factors: combine task performance and workplace well-being	1,192.59	51	0.74	0.66	0.262
Model 3	Two factors: combine CFSB and coworker relationship, task performance and workplace well-being	2,191.20	53	0.51	0.38	0.351

N = 327. CFSB stands for coworker feedback-seeking behavior.

Descriptive statistics results are shown in Table 2. As shown, the correlation between feedback-seeking from coworkers and task performance (*r* = *0.27, p*< *0.01*) and workplace well-being (*r* = *0.16, p*< *0.01*) was positive. The correlation between feedback-seeking from coworkers and coworker relationship (*r* = *0.35, p*< *0.01*) was positive. In addition, the correlation between coworker relationship and task performance (*r* = *0.42, p*< *0.01*) and workplace well-being (*r* = *0.31, p*< *0.01*) was positive. These results provided initial support for Hypotheses 1a to 3b.

**Table 2:** Means, standard deviations, reliabilities, and correlations among study variables.

	**Variables**	**Mean**	**SD**	**1**	**2**	**3**	**4**	**5**	**6**	**7**
1	Gender	1.96	0.20							
2	Age	34.32	9.80	−0.10						
3	Education	2.84	0.39	0.16**	0.22**					
4	CFSB	3.66	0.76	0.01	−0.27**	−0.07	**(0.90)**			
5	Coworker relationship	4.20	0.64	0.04	−0.12*	−0.06	0.35**	**(0.93)**		
6	Task performance	4.51	0.52	0.12*	−0.03	0.04	0.27**	0.42**	**(0.96)**	
7	Workplace well-being	4.13	0.66	−0.03	0.07	−0.06	0.16**	0.31**	0.40**	**(0.94)**

N = 327. CFSB stands for coworker feedback-seeking behavior. Cronbach's alpha reliabilities are in parentheses on the diagonal, *p < 0.05, **p < 0.01.

### Mediating effects test

Hypotheses 1a to 1b proposed the direct positive relationship between feedback-seeking from coworkers and task performance, and workplace well-being. As shown in Table 3, after considering the control variables, feedback-seeking from coworkers was positively related to task performance (*B* = *0.20, SE* = *0.04, p*< *0.001, Model 1*) and workplace well-being (*B* = *0.17, SE* = *0.05, p*< *0.001, Model 1*), thus supporting Hypotheses 1a and 1b. Hypothesis 2 assumed the relationship between feedback-seeking from coworkers and coworker relationship. Results showed that feedback-seeking from coworkers was positively related to coworker relationship (*B* = *0.29, SE* = *0.05, p*< *0.001, Model 2*), providing support for Hypothesis 2.

**Table 3:** Main effects and mediating effects (M1 and M2).

**Predictors**	**Task performance**		**Workplace well-being**		**Coworker relationship**		**Task performance**		**Workplace well-being**	
	**Model 1**				**Model 2**					
	***B***	***SE***	***B***	***SE***	***B***	***SE***	***B***	***SE***	***B***	***SE***
Intercept	2.97***	0.37	3.59***	0.48	3.06***	0.45	2.04***	0.37	2.69***	0.49
Gender	0.32*	0.14	−0.01	0.19	0.14	0.17	0.27*	0.13	−0.05	0.18
Age	0.00	0.00	0.01*	0.00	−0.00	0.00	0.00	0.00	0.01**	0.00
Education	0.04	0.07	−0.14	0.10	−0.06	0.09	0.06	0.07	−0.12	0.09
CFSB	0.20***	0.04	0.17***	0.05	0.29***	0.05	0.11**	0.04	0.08^†^	0.05
Coworker relationship							0.30***	0.04	0.30***	0.06
Residual Variance	0.25***	0.02	0.41***	0.03	0.36	0.03	0.22***	0.02	0.38***	0.03
R^2^	0.08		0.04		0.12		0.19		0.11	

N = 327. CFSB stands for coworker feedback-seeking behavior, *p < 0.05, **p < 0.01, ***p < 0.001. ^†^represents p < 0.1.

Hypotheses 3a and 3b posited the indirect effect of feedback-seeking from coworkers on task performance and workplace well-being *via* coworker relationship. Our results showed that coworker relationship was positively related to task performance (*B* = *0.30, SE* = *0.04, p*< *0.001, Model 2*) and workplace well-being (*B* = *0.30, SE* = *0.06, p*< *0.001, Model 2*) after controlling demographics and feedback-seeking from coworkers, which provide initial support for Hypotheses 3a and 3b. To confirm the mediating effect of coworker relationship, we applied the MODEL CONSTRAINT command in Mplus 8 to calculate the indirect effect. Our results showed that the indirect effect of feedback-seeking from coworkers on task performance through coworker relationship was 0.089 (*SE* = *0.02, p*< *0.001, 95% CI [0.052, 0.125]*), and the indirect effect of feedback-seeking from coworkers on workplace well-being through coworker relationship was 0.086 (*SE* = *0.02, p*< *0.001, 95% CI [0.044, 0.128]*), thus fully supporting Hypotheses 3a and 3b.

### Moderating effects test

Hypothesis 4 proposed the moderating role of coworker relationship in the relation between feedback-seeking from coworkers and task performance. As shown in Table 4, the interaction term (feedback-seeking from coworkers × coworker relationship) was negatively and significantly related to task performance (*B* = −*0.13, SE* = *0.05, p*< *0.05, Model 3*), thus providing support for Hypothesis 4.

**Table 4:** Moderating effects of coworker relationship (M3).

	**Task**		**Workplace**	
	**performance**		**well-being**	
	***B***	***SE***	***B***	***SE***
Intercept	2.12***	0.37	2.78***	0.49
Gender	0.27*	0.13	−0.06	0.18
Age	0.00	0.00	0.01**	0.00
Education	0.06	0.07	−0.12	0.09
CFSB	0.13***	0.04	0.11*	0.05
Coworker relationship	0.27***	0.05	0.26***	0.06
CFSB × coworker relationship	−0.13*	0.05	−0.15*	0.07
Residual variance	0.21***	0.02	0.37***	0.03
*R* ^2^	0.23		0.14	

N = 327. CFSB stands for coworker feedback-seeking behavior, *p < 0.05, **p < 0.01, ***p < 0.001.

Similarly, Hypothesis 5 proposed the moderating role of coworker relationship in the relation between feedback-seeking from coworkers and workplace well-being. As shown in Table 4, the interaction term (feedback-seeking from coworkers × coworker relationship) was negatively and significantly related to workplace well-being (*B* = −*0.15, SE* = *0.07, p*< *0.05, Model 3*), thus providing support for Hypothesis 5.

**Figure 2:**
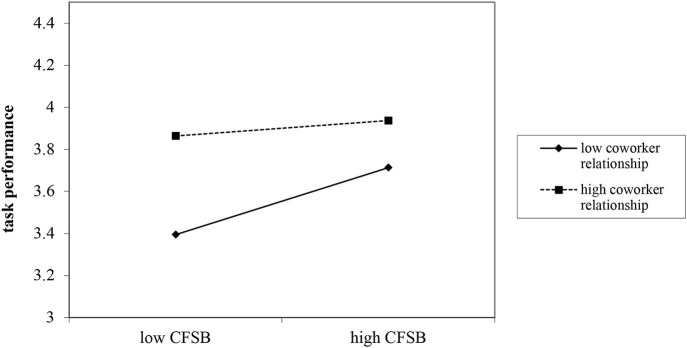
The moderating role of coworker relationship on the coworker feedback-seeking behavior (CFSB) and task performance relationship.

**Figure 3:**
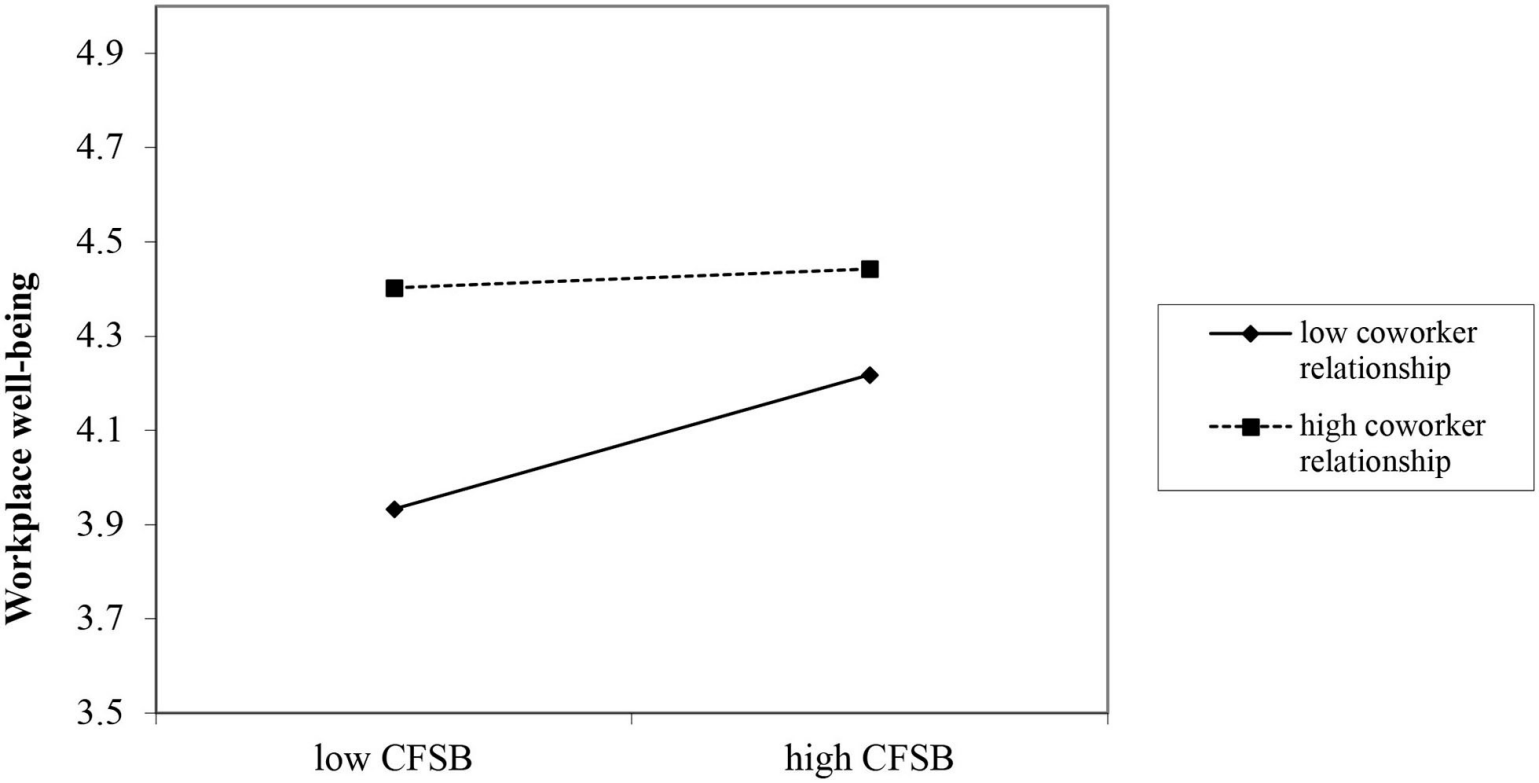
The moderating role of coworker relationship on the coworker feedback-seeking behavior (CFSB) and workplace well-being relationship.

To better interrupt the moderating effect of coworker relationship, we defined high and low levels of coworker relationship as plus and minus one SD from the mean (Cohen and Cohen, 1983). As shown in Figure 2, the relationship between feedback-seeking from coworkers and task performance was stronger for teachers with lower (i.e., −1 SD) coworker relationship (*simple slope* = *0.21, p*< *0.001*) rather than the teachers with higher (i.e., +1 SD) coworker relationship (*simple slope* = *0.05, ns*.). Analogously, Figure 3 shows that the relationship between feedback-seeking from coworkers and workplace well-being was stronger for teachers with lower (i.e., −1 SD) coworker relationship (*simple slope* = *0.21, p*< *0.01*) rather than the teachers with higher (i.e., +1 SD) coworker relationship (*simple slope* = *0.01, ns*.).

## Discussion

Feedback-seeking behavior scholars have shifted their focus from seeking feedback from supervisors to coworkers and explored its effect on performance (Wu et al., 2014; De Stobbeleir et al., 2020) as well as the potential mechanisms from the cognitive perspective (i.e., role clarity) (Whitaker et al., 2007). Moving on to these studies, we theorized and examined the roles of coworker relationship based on social exchange theory in the feedback-seeking process. Our results indicated that coworker relationship played mixed roles in the coworker feedback-seeking process: on the one hand, it transformed the positive effect of coworker feedback-seeking behavior on task performance and workplace well-being; on the other hand, it hindered the positive effect of coworker feedback-seeking on task performance and workplace well-being.

### Theoretical implications

Our study provides several theoretical contributions. First, we contribute to the feedback-seeking behavior literature by shifting the focus to peers' feedback-seeking and examining one possible mechanism of this behavior. Existing research mostly explored the feedback-seeking between employees and supervisors (Ashford et al., 2016), ignoring the possible benefits of peer feedback-seeking (with exceptions: Wu et al., 2014; De Stobbeleir et al., 2020). However, these two studies only established the direct effect of coworker feedback-seeking behavior and work outcomes (e.g., job performance). We move on this research line by identifying one transformed mechanism behind this behavior (i.e., coworker relationship). Our results indicate that seeking feedback from peers is positively related to task performance and workplace well-being *via* increased coworker relationship.

Second, we focus on the relational aspect of feedback-seeking behavior and expand its outcomes. As De Stobbeleir et al. (2020) proposed, peer feedback-seeking behavior was different from other types of proactive behaviors. In detail, unlike behaviors such as job crafting (Zhang and Parker, 2019), which is toward individuals themselves, and organizational citizenship behavior, which is toward the organization, peer feedback-seeking behavior is more relationally orientated (De Stobbeleir et al., 2020, P. 316). Thus, it will be helpful for creating, maintaining, and improving relationships among colleagues. By testing the relationship between coworker feedback-seeking behavior and coworker relationship, we answered De Stobbeleir et al.'s (2020) call for further empirical exploration of this “relational proactive behavior.” Furthermore, based on the relational and interactive nature of feedback-seeking behavior, we expand prior research, which primarily focused on the “cold” outcomes (e.g., performance) of feedback-seeking behavior (e.g., Gong et al., 2017; Lam et al., 2017), by investigating its influence on the “hot” outcomes (i.e., workplace well-being).

Third, we extend the use of social exchange theory by considering the dual effect of social exchange embedded in peers' feedback-seeking. Extant research has contended that social exchange was an effective way for employees to replenish resources and then help conquer the adverse situation. For example, previous studies have yielded the mitigating effect of coworker relationship on negative workplace experiences (e.g., workplace loneliness and workplace anxiety) (McCarthy et al., 2016; Jung et al., 2021), and the expanding effect of social exchange on positive workplace experiences (e.g., positive leadership) (Zhang et al., 2021). Beyond the positive effects, extant studies have focused on the potential negative effect of high-quality social exchange. For instance, Xing et al. (2021) found that high-quality relationships would strengthen the effect of leaders' negative feedback on employees' shame, as employees would feel guilty in the high-quality LMX context after receiving negative feedback. Following this research line, we focused on the effectiveness and quality of social exchange and explored both the positively transforming effect of coworker relationship and the hindering roles of coworker relationship. Our findings indicated that coworker feedback-seeking behavior and coworker relationship work simultaneously, and they compete and compensate each other. By doing so, we contribute to social exchange theory by combining both the positive and negative effects of social exchange in feedback-seeking behavior. We also echo Lam et al.' (2017) study, which demonstrated the side effect of LMX on seeking feedback from supervisors, as well as Parker et al.'s (2019) review, which contended that proactivity (e.g., feedback-seeking behavior) was not always wise.

### Practical implications

The current research has some practical implications. First, our findings indicated that peers are also important feedback sources, and seeking feedback from them helps employees improve task performance and well-being. Therefore, managers should encourage employees to perform this behavior proactively. This could be achieved by loosening the costs embedded in feedback-seeking behavior. For example, managers or organizations could increase the psychological safety atmosphere (Lan et al., 2020), or establish a supportive feedback environment to promote this behavior (Whitaker et al., 2007).

Second, we found a positive relationship between coworker feedback-seeking and coworker relationship. This provides managers with insights on how to improve and manage employee relations. In other words, managers could establish a harmonious team or organizational atmosphere by encouraging communication and interaction between colleagues, especially encouraging employees to seek feedback from peers (De Stobbeleir et al., 2020).

Third, our research also revealed the dark side of coworker relationship. It is notable that seeking feedback from coworkers with low-quality relationship rewards more, as the high-quality relationship would undermine the values of peer feedback. We recommend that seekers should expand the scope of feedback sources, and focus on the peers they usually less communicate and interact with each other (Ashford et al., 2016). By asking for feedback from this group of coworkers, employees were more likely to receive novel and valuable ideas. This could be done by improving employees' interpersonal skills and the ability to identify the values and accuracy of feedback (Anseel and Lievens, 2009).

### Limitations and directions

Our study has some limitations. First, the cross-sectional design in our study makes it difficult to predict the causal relations among our studied variables. While we indicated that coworker feedback-seeking behavior improved coworker relationship, reverse causality is also possible in this relationship (Lan et al., 2020). Future studies will be encouraged to examine this relationship using longitudinal data to examine the relationship between coworker feedback-seeking behavior and coworker relationship.

Second, we only explored the seekers' feelings and work outcomes, while neglecting the potential reactions and results from the feedback source perspective (i.e., the coworker). Research has indicated that feedback sources' experiences and reactions toward feedback-seeking behavior also influenced the effectiveness of this behavior (Minnikin et al., 2021). Therefore, an inversed U-shape relationship may exist between coworker feedback-seeking and coworker relationship. Specifically, because responding to others' feedback-seeking consumes time and energy, coworkers may respond actively in the early stage but may be less responsive to this behavior as the frequency increases. Accordingly, the relationship will be positive at first and then turn negative with the increase in feedback-seeking behavior. Future studies would be helpful to investigate coworker feedback-seeking behavior from both the seeker and source perspectives.

Third, we only explained the positive benefits of coworker feedback-seeking behavior following the relationship perspective. Although peer feedback-seeking behavior is a kind of “relational proactivity” (De Stobbeleir et al., 2020), other perspectives such as emotional reactions in the feedback-seeking process are also helpful to further understanding the interactive process (Methot et al., 2021). Future studies thus are necessary to introduce more theoretical perspectives to explore the mechanisms of coworkers' feedback-seeking behavior.

Finally, we tested our theoretical model using participants from kindergartens, which were mainly composed of women. This will constrain the external validity of the studied results to some extent. Although the current sample meets the characteristics of teamwork, and gender has been taken as one of the control variables, we encourage future studies to explore our model across different samples.

## Conclusion

Taken together, following social exchange theory, the current study reveals that coworker feedback-seeking behavior benefits the seekers (i.e., improved task performance and workplace well-being) by improving coworker relationship, and at the same time, coworker relationship hinders the positive effects of coworker feedback-seeking behavior on task performance and workplace well-being. By applying a relational perspective, we explain how and when individuals could benefit most from coworker feedback-seeking behavior. Our findings thus provide insights into how to manage feedback-seeking behavior in the workplace.

## Data availability statement

The original contributions presented in the study are included in the article/supplementary material, further inquiries can be directed to the corresponding author/s.

## Ethics statement

The studies involving human participants were reviewed and approved by Business School, Beijing Normal University. The patients/participants provided their written informed consent to participate in this study.

## Author contributions

WZ and JQ contributed to the conception and design of the study. HY organized the database. WZ and HY performed the statistical analysis and completed the first draft of the manuscript. All authors contributed to the article and approved the submitted version.

## Funding

This research was supported by the National Natural Science Foundation of China (71871025).

## Conflict of interest

The authors declare that the research was conducted in the absence of any commercial or financial relationships that could be construed as a potential conflict of interest.

## Publisher's note

All claims expressed in this article are solely those of the authors and do not necessarily represent those of their affiliated organizations, or those of the publisher, the editors and the reviewers. Any product that may be evaluated in this article, or claim that may be made by its manufacturer, is not guaranteed or endorsed by the publisher.

## Footnotes

^1^We compared the demographics of the final sample (327 teachers) with the initial sample (546 teachers) using an independent sample T-test. Results showed that there was no significant difference between these two samples [*t*_(871)_ = *1.22, p* = *0.22* for gender; *t*_(871)_ = *1.56, p* = *0.12* for age; *t*_(871)_ = *1.27, p* =*0.20* for education].
